# Decreased perifoveal ganglion cell complex thickness - a first sign for macular damage in patients using hydroxychloroquine

**DOI:** 10.22336/rjo.2023.26

**Published:** 2023

**Authors:** Sümeyye Burcu Elpeze Agcayazi, Vuslat Gurlu, Goksu Alacamli

**Affiliations:** * Ophthalmology Department, Istanbul Training and Research Hospital, Istanbul, Turkey; ** Ophthalmology Department, Ekol Hospital, Edirne, Turkey; *** Ophthalmology Department, Trakya University Faculty of Medicine, Education and Research Hospital, Edirne, Turkey

**Keywords:** hydroxychloroquine, Optical Coherence Tomography, ganglion cell complex thickness, Retinal Nerve Fiber Layer

## Abstract

**Aim:** To examine ganglion cell complex (GCC) thickness detected by optical coherence tomography (OCT) in patients using hydroxychloroquine (HCQ), without any structural and functional macular changes to evaluate the initial symptoms of macular toxicity for early diagnosis before clinical evaluation.

**Methods:** Eighty eyes of forty patients (Group 1) and forty eyes of twenty healthy volunteer persons (Group 2) were included in the study. Detailed ophthalmologic and mydriatic fundus examination were applied to all patients and volunteers (controls). Spectral domain OCT, visual field (VF) and color vision test were performed. Measurements of macula thickness, GCC thickness (involving nerve fiber layer, ganglion cell layer and inner plexiform layer) and peripapillary retinal nerve fiber layer (RNFL) were performed with OCT. Patients with retinal pigment epithelial changes, VF paracentral scotoma and defected color vision were excluded from the planned study.

**Results:** Perifoveal GCC layer thickness in all quadrants was significantly thinner in group 1 compared to group 2 (p=0.017, p=0.001, p=0.019, p=0.001). The mean global inferior hemifield and nasal quadrant RNFL thickness were lower than in the control groups (p=0,012, p=0,009, p=0,005, respectively).

**Conclusion:** Changes in the thickness of nerve fiber layer and ganglion cell layer detected by optical coherence tomography can be thought to be used as a diagnostic aid for the early diagnosis of hydroxychloroquine-toxic maculopathy

**Abbreviations: **GCC = Ganglion cell complex, OCT = Optical coherence tomography, HCQ = Hydroxychloroquine, BCVA = Best-corrected visual acuity, IOP = Intraocular pressure, VF = Visual field, RNFL = Retinal nerve fiber layer, SD OCT = Spectral-domain optical coherence tomography, mfERG = Multifocal electroretinogram, FAF = Fundus autofluorescence, IS/ OS = Inner segment-outer segment junction, SITA = Swedish Interactive Threshold Algorithm, RA = Rheumatoid arthritis, SLE = Systemic lupus erythematosus, SS = Sjogren syndrome

## Introduction

Hydroxychloroquine (HCQ) retinopathy is a toxic retinopathy that occurs in 0.5% of patients treated with chronic HCQ for various rheumatic diseases [**1**]. Clinical symptoms of HCQ retinopathy are: visual acuity lowering and defects in color vision, annular scotoma and accompanying bull’s-eye maculopathy [**2**,**3**]. Early diagnosis is important because after the toxic retinopathy appearance, drug discontinuation does not reverse the functional loss [**4**,**5**]. 

Visual field examination and ophthalmoscopy are the routine examination methods for monitoring HCQ toxicity [**6**]. However, the diagnoses determined with these methods include only the functional and structural changes. Thus, new sensitive objective tests are recommended for screening and early diagnosis. After revision in 2016, the American Academy of Ophthalmology recommended that the beginning of cross-examination for monitoring HCQ toxicity was automatic visual field test and spectral domain optical coherence tomography (SD-OCT). Fundus autofluorescence (FAF) can detect focal damage and multifocal electroretinogram (mfERG) and can be useful for objective evidence for visual acuity. Current scans should show us the retinopathy before it is seen by ophthalmoscopy [**7**]. Recently, several studies on the validity and sensitivity of OCT, microscopic perimetry, and FAF have produced varied results. Nevertheless, its sensitivity varies from case to case. OCT images showed that different layers were affected parallel to the seriousness of retinopathy [**8**,**9**]. Microperimetric alterations accompanying retinopathy have been described [**10**]. Marmor et al. showed that sensitivity of early diagnosis toxic retinopathy tests varies in all cases, and hydroxychloroquine leads early parafoveal loss of the outer segment on SD-OCT, with the initial deteriorations, usually seen in the inferotemporal quadrant [**11**]. Also, visual field examination and OCT were suggested by Marmor et al. for screening retinopathy [**7**].

According to the OCT results reported in literature, perifoveal layer was shown to be thinner in the inner retinal layer in HCQ of patients without clinical ophthalmic findings [**12**,**13**]. However, previous studies included a small number of subjects examined. In addition to that, the effect of the cumulative dose (known as the most critic risk factor for the development of retinopathy) on the thickness of the parafoveal segment has not been evaluated before.

The objective of the study was to make the comparison of the OCT Ganglion Cell Complex (GCC) thickness between the patients and the control group, who had no structural and functional changes, also to examine the effect of HCQ cumulative dose on the GCC thickness.

## Materials and methods

The data of both patients and controls, who were examined in our outpatient clinic with different indications between May 2012 and August 2019, were prospectively analyzed. Eighty eyes of forty patients (Group 1) and forty eyes of twenty healthy subjects (Group 2) were added to the study. All procedures and steps for the study were supported by the hospital’s institutional review board and accommodated to the characteristics of the Helsinki Declaration.

Both patients and volunteers (controls), who had spherical refractive error equal or less than ± 6.0 D and cylinder refractive error equal or less than ± 3 D, and intraocular pressure (IOP) equal or less than 21 mm Hg were included to the data collection of the study. Patients who had any media opacities (originating from cornea or lens) history of drug therapy, retinal disease, diagnose of glaucoma, liver or kidney or any ocular disorder, ocular surgery or trauma history, micro changes in the inner segment-outer segment junction, which is called IS/ OS, in the localization of the fovea, or interruption or loss in the OCT, were excluded from the study. The medical history of all the patients (doses of hydroxychloroquine use and the total amount of time - years - to calculate the cumulative dose) was taken. Best-corrected visual acuity (BCVA), IOP measurement (NT-4000 Auto Non-Contact Tonometer, Nidek, Japan), slit lamp and mydriatic fundus examination were applied. Spectral domain OCT (RS-3000 Lite, Nidek, Japan), visual field (VF) test, (Humphrey Field Analyzer using the 10-2 Swedish Interactive Threshold Algorithm (SITA)-Standard algorithm of the Humphrey perimeter (HFA-II Carl Zeiss Meditec) and color vision test (Farnsworth-Munsell 100 Hue) were added to the full ophthalmological examination. 

Patients who were diagnosed with retinal pigmentary epithelium changes (6 eyes), paracentral scotoma in VF (6 eyes) and color vision defects (6 eyes) were excluded from the study. Some eyes had 2 or 3 simultaneous exclusions. A total of 72 eyes were involved in the study.

Macula thickness, ganglion cell complex (GCC) thickness (nerve fiber layer, ganglion cell layer and inner plexiform layer) and peripapillary retinal nerve fiber layer (RNFL) were measured with OCT. The parameters used for macular thickness statistical analysis were: 1 mm central macular area, 1-3 mm paramacular area and 3-6 mm perifoveal macular area. Parafoveal and perifoveal area of the macula were divided to superior, inferior, nasal, and temporal quadrants. Parameters for the statistical analysis of the GCC thickness were: 1-3 mm parafoveal macular area and 3-6 mm perifoveal macular area. Both the thickness of 1-3 mm parafoveal macular area and 3-6 mm perifoveal macular area of GCC were divided to superior nasal, superior temporal, inferior nasal, and inferior temporal quadrants. Disk map protocol was used for the evaluation of peripapillary RNFL. Peripapillary region was divided into the total, superior hemifield, inferior hemifield, superior, nasal, inferior and temporal quadrants, similar to macular and GCC thickness calculation.

SPSS 15.0 (SPSS Inc., Chicago, IL) was used for statistical analysis. The normality assumption was analyzed using Kolmogorov-Smirnov test. Independent Sample T test was used for the comparison of the normally distributed data. Mann-Whitney U Test was used for comparisons of the data that were not normally distributed. Pearson Correlation Analysis was performed to determine the relationship between parameters. P<0.05 was defined as statistical significance.

## Results

Our study included eighty eyes of the 40 patients (38 females, mean age 48,4 ± 14,4 years) and forty eyes of the 20 healthy, volunteer control subjects (19 females, mean age 49 ± 12,8 years). No significant differences were shown in the demographic data between the groups (p>0.05). We investigated 22 rheumatoid arthritis (RA) patients, 14 Systemic lupus erythematosus (SLE) patients, and 4 Sjogren syndrome (SS) patients using HCQ. The mean duration of use of HCQ in group 1 was 6.4 ± 5.1 (1-20) years. The mean cumulative dose of HCQ usage was 504.88 ± 387.4 gr. 

The corrected visual acuity of all patients was 20/ 20. Slit lamp and ophthalmoscopy were normal in all cases. Visual field analysis and color visual field tests were good in both groups. None of the subjects had evidence of any damage to the IS/ OS junction seen by OCT. 

We could not see any significant difference between patients and controls in terms of central macular thickness, parafoveal macular thickness and perifoveal macular thickness (**Table 1**). Patients and controls did not show any statistically significant difference in the thickness of the parafoveal ganglion cell complex layer. However, in the patient group, perifoveal GCC layer thickness was statistically significantly thinner, when compared to the control group, in superior nasal, superior temporal, inferior nasal, inferior temporal quadrants respectively (p=0,017, p=0,001, p=0,019, p=0,001, respectively) (**Table 2**). The mean global, inferior hemifield and nasal quadrant RNFL thickness measurements were thinner than the control group (p=0,012, p=0,009, p=0,005) (**Table 3**).

**Table 1:** Central, perifoveal and parafoveal macular thickness (μ) of the patients and control groups

Macula	Group 1	Group 2	p*
Fovea	268,0 ± 16,2	268,5 ± 17,6	0,967
Parafovea superior	339,4 ± 18,6	339,8 ± 11,6	0,365
Parafovea inferior	333,8 ± 20,6	337,4 ± 11,1	0,944
Parafovea nasal	337,6 ± 21,4	340,6 ± 9,8	0,576
Parafovea temporal	324,5 ± 17,1	325,7 ± 8,9	0,644
Perifovea superior	299,8 ± 15,9	302,7 ± 18,0	0,885
Perifovea inferior	292,0 ± 17,5	298,6 ± 16,8	0,120
Perifovea nasal	313,0 ± 22,2	315,4 ± 16,3	0,900
Perifovea temporal	286,8 ± 14,4	288,0 ± 15,2	0,969

**Table 2:** Parafoveal and perifoveal GCC thickness (μ) of the patients and control groups

GCC	Group 1	Group 2	p*
Parafovea superior nasal	115,7 ± 14,1	116,9 ± 10,3	0,692
Parafovea superior temporal	109,1 ± 10,0	109,0 ± 10,1	0,845
Parafovea inferior nasal	114,5 ± 13,7	116,3 ± 9,9	0,936
Parafovea inferior temporal	108,4 ± 12,8	109,3 ± 11,5	0,796
Perifovea superior nasal	108,3 ± 12,2	114,1 ± 11,7	0,017
Perifovea superior temporal	89,5 ± 6,9	94,1 ± 8,2	0,001
Perifovea inferior nasal	109,9 ± 12,4	116,6 ± 11,2	0,019
Perifovea inferior temporal	92,5 ± 10,8	98,2 ± 6,6	0,001
GCC = Ganglion cell complex			

**Table 3:** Retina nerve fiber layer thickness (μ) measurements

RNFL	Group 1	Group 2	p*
Total	100,03 ± 11,8	106,3 ± 11,4	0,012
Superior hemifield	102,3 ± 12,3	107,4 ± 15,7	0,077
Inferior hemifield	110,8 ± 114,4	104,5 ± 12,1	0,009
Superior	124,1 ± 16,2	130,9 ± 21,7	0,182
Inferior	128,7 ± 22,1	135,0 ± 20,0	0,236
Nasal	78,1 ± 14,0	85,7 ± 14,9	0,005
Temporal	65,6 ± 11,3	68,7 ± 14,4	0,199
RNLF = retinal nerve layer fiber			

HCQ cumulative dose and perifoveal GGC thickness showed significant correlation in superior temporal, inferior nasal, inferior temporal quadrants (p=0,009, p=0,012, p=0,001 respectively) (**Table 4**).

**Table 4:** Correlation analysis between the cumulative dose of HCQ and GGC thickness (μ) parameters

GCC	r	p
Parafovea superior nasal	0,123	0,181
Parafovea superior temporal	0,10	0,917
Parafovea inferior nasal	-0,73	0,425
Parafovea inferior temporal	-0,104	0,257
Perifovea superior nasal	-0,84	0,361
Perifovea superior temporal	-0,238	0,009
Perifovea inferior nasal	-0,228	0,012
Perifovea inferior temporal	-0,295	0,001

A significant correlation between the HCQ cumulative dose and peripapillary RNFL thickness in superior hemifield, superior and nasal quadrants (p=0,021, p=0,038, p=0,002, respectively) was observed (**Table 5**).

**Table 5:** Correlation analysis between the cumulative dose of HCQ and RNFL thickness (μ) parameters

RNFL	r	p
Total	-0,282	0,385
Superior hemifield	-0,211	0,021
Inferior hemifield	-0,084	0,361
Superior	-0,190	0,038
Inferior	-0,076	0,409
Nasal	-0,276	0,002
Temporal	-0,071	0,440

## Discussion

Recently, antimalarials have been used for systemic autoimmune and inflammatory diseases [**14**]. Chloroquine (CQ) has more side effects than hydroxychloroquine (HCQ), so the use of CQ is limited [**15**]. Due to their melanotropic properties, antimalarials accumulate in the retinal pigment epithelium (RPE), ciliary body and iris [**16**]. Some of the ocular side effects of antimalarial drugs are keratopathy, ciliary body pathologies, lens opacities, like cortical cataract and retinopathy. However, among the many side effects, the most common is toxic maculopathy [**3**].

Clinical studies have shown that the first pathological changes occur in retinal ganglion cells in the patients using of HCQ [**17**]. Antimalarial drugs disrupt the lysosomal functions and thus lipofuscin accumulates in RPE and causes toxic damage, which can be seen by fundoscopy, during early stages [**18**]. Target-board maculopathy (bull’s-eye maculopathy), which is characterized by concentric hypopigmentation surrounded by a hyperpigmentation ring, develops in later stages. During late toxicity, it can occur or progress even without medication. In the initial phase, toxicity is reversible after drug discontinuation [**19**]. Therefore, early detection of toxicity is very important. However, since normal fundus images can occur in the initial stages, more detailed testing is needed to diagnose toxicity at this stage. Retinal toxicity is known to be a serious side effect in the patients using HCQ and can be irreversible. Although the incidence of HCQ maculopathy is low, permanent or progressive effects can be observed even after HCQ is stopped [**12**]. The mechanism underlying HCQ maculopathy remains an ophthalmic mystery. Age, more than 5 years of treatment, liver or kidney disease, and increased drug use, as cumulative dose, were the most important factors previously reported [**7**]. Cumulative dose, rather than the daily dose, has been suggested to be more influential on toxicity, but it is still clearly explained. Because of that, individual HCQ doses are recommended [**7**,**20**].

There are no specific diagnostic criteria for the detection of the early signs of macular damage for the early diagnosis before macular toxicity occurs [**12**]. Previous studies have reported several ways to detect damage at an early stage: visual acuity, multifocal electroretinogram, fundus autofluorescence, near infrared autofluorescence, microperimetry and OCT [**12**]. In the present study, we used spectral domain OCT to examine early signs of macular damage. Animal studies showed that the first pathological change is the accumulation of cytoplasmic granules in the ganglion cells and progression to ganglion cell degeneration. Photoreceptor degeneration and RPE cell degeneration occur at the later stages [**13**]. IS/ OS junction changes and thinning of the outer nuclear layer of retina were shown in previous studies [**21**]. Changes in the outer retinal layer have also been observed but not reported as an early indicator of toxicity [**8**,**12**]. Stephen et al. showed the SD-OCT results of 2 patients. They had been taking HCQ medication long before the clinic of visual impairment. In areas correlated with visual defects, they found IS/ OS degradation and preservation of RPE. They observed a “moth-eaten” view in the IS/ OS layer in patients without visible defects [**8**].

We found that, perifoveal ganglion cell complex layer in the patient group was significantly thinner than in the control group. On the other hand, thinning of central, parafoveal and perifoveal macular area was not found in the patients’ group. Pasdika et al. pointed out conspicuous thinning of the inner retinal layer in patients without ophthalmologic changes, which showed significant thinning of the inner retinal layer in the perifoveal region [**13**]. The major characteristics were the full integrity of the IS/ OS junction of all our patients and each quadrant was investigated separately. The thinning in all perifoveal quadrants was observed. Moreover, superior temporal and inferior temporal quadrants were more significantly affected. Yiğit et al. showed thinning of the inner retina only in the inferior quadrants in patients without clinical toxicity [**12**]. In our study, the thinning of all quadrants may be related to longer HCQ intake period (25% of our patients’ intake was over a period of more than 10 years) than in the previous studies. 

In peripapillary RNLF analysis, we found that the global, inferior hemifield and nasal quadrant retina nerve fiber layers were thinner in the patients’ group. Controversially, Yılmaz and Saatci [**22**] found no difference in RNLF thickness between patients using antimalarial drugs and healthy control groups. Pashadika et al. observed thinning in the nasal quadrants of the patients without fundus changes in the peripapillary RNLF analysis [**13**].

Kan et al. included ninety eyes of ninety patients taking HCQ for at least 5 years in their study. Also, patients had lower macular GC-IPL thickness than the controls [**23**].

Min Gyu Lee et al. found that macular GCC thickness did not show a clear association with HCQ use, but some patients, particularly those with HCQ retinopathy or high doses, had thinner GC-IPL [**24**]. Their results were parallel to our study. 

Similar to our study, the results of Bulut et al. showed that patients taking HCQ had thinner GCC at early stage. According to these results, measuring GCC thickness could become an important target of HCQ screening [**25**].

Uslu et al. found a thinner and infratemporal macular GC-IPL thickness in patients receiving hydroxychloroquine compared to controls [**26**]. The results were similar to the ones in our study.

Salam et al. found that all OCT measurements were decreased (p<0.001) in HCQ patients, including GCC thickness, compared to the control group, which were similar to our study [**27**].

Kurna et al. found that all GCC parameters detected by OCT were decreased in HCQ patients [**28**].

The limitation of the study was that we did not use the data of the microscopic perimetry, which can be a useful device for the early sign of the toxicity that can be found immediately, but only in high places. Among the strengths of our work were the prospective design, the large number of patients who had no clinical or diagnostic changes, and RPE or IS/ OS junction morphological changes in OCT.

## Conclusion

As a conclusion, this prospective study pointed out that the cumulative usage of HCQ led to an early stage of the perifoveal thinning of the GGC process. OCT is a reliable and repeatable method for the monitorization of the HCQ toxicity.

**Conflict of Interest statement**

The authors state no conflict of interest.

**Informed Consent and Human and Animal Rights statement**

Informed consent has been obtained from all individuals included in this study.

**Authorization for the use of human subjects**

Ethical approval: The research related to human use complies with all the relevant national regulations, institutional policies, is in accordance with the tenets of the Helsinki Declaration, and has been approved by the review board of Trakya University Education and Research Hospital, Edirne, Turkey.

**Acknowledgements**

None.

**Sources of Funding**

None.

**Disclosures**

None.

## References

[1] Mavrikakis I, Sfikakis PP, Mavrikakis E, Rougas K, Nikolaou A, Kostopoulos C, Mavrikakis M (2003). The incidence of irreversible retinal toxicity in patients treated with hydroxychloroquine: a reappraisal. Ophthalmology.

[2] Tehrani R, Ostrowski RA, Hariman R, Jay WM (2008). Ocular toxicity of hydroxychloroquine. Semin Ophthalmol.

[3] Bernstein HN (1983). Ophthalmologic considerations and testing in patients receiving long term antimalarial therapy. Am J Med.

[4] Rynes RI, Bernsteın HN (1993). Ophthalmologic safety profile of antimalarial drugs. Lupus.

[5] Easterbrook M (2002). Screening for antimalarial toxicity: Current concepts. Can J Ophthalmol.

[6] Marmor MF, Carr RE, Easterbrook M, Farjo A, Mieler M (2002). Recommendations on screening for chloroquine and hydroxychloroquine retinopathy: a report by the American Academy of Ophthalmology. Ophthalmology.

[7] Marmor MF, Kellner U, Lai TY, Melles RB, Mieler WF (2016). American Academy of Ophthalmology. Recommendations on Screening for Chloroquine and Hydroxychloroquine Retinopathy (2016 Revision). Ophthalmology.

[8] Stepien KE, Han DP, Schell J, Godara P, Rha J, Carrol J (2009). Spectral-domain optical coherence tomography and adaptive optics may detect hydroxychloroquine retinal toxicity before symptomatic vision loss. Trans Am Ophthalmol Soc.

[9] Eric C, David MB, Matthew SB (2010). Spectral domain optical coherence tomography as an effective screening test for hydroxychloroquine retinopathy (the “flying saucer” sign). Clin Ophthalmol.

[10] Martínez-Costa L, Victoria Ibañez M, Murcia-Bello C, Epifanio I, Verdejo-Gimeno C, Beltrán-Catalán E, Marco-Ventura P (2013). Use of microperimetry to evaluate hydroxychloroquine and chloroquine retinal toxicity. Can J Ophthalmol.

[11] Marmor MF (2012). Comparison of screening procedures in hydroxychloroquine toxicity. Arch Ophthalmol.

[12] Yıgıt U, Tugcu B, Tarakcioglu HN, Celık S (2013). Spectral domain optical coherence tomography for early detection of retinal alterations in patients using hydroxychloroquine. Ind J Ophthalmol.

[13] Pasadhika S, Fishman GA, Choi D, Shahidi M (2010). Selective thinning of the perifoveal inner retina as an early sign of hydroxychloroquine retinal toxicity. Eye.

[14] Bell CL (1983). Hydroxychloroquine sulfate in rheumatoid arthritis: long-term response rate and predictive parameters. Am J Med.

[15] Nylander U (1966). Ocular damage in chloroquine therapy. Acta Ophthalmologica.

[16] Mackenzıe AH (1983). Pharmacologic actions of 4-aminoquinoline compounds. Am J Med.

[17] Mahon GJ, Anderson HR, Gardiner TA, McFarlane S, Archer DB, Stitt AW (2004). Chloroquine causes lysosomal dysfunction in neural retina and RPE: implications for retinopathy. Curr Eye Res.

[18] Gonasun LM, Potts AM (1974). In vitro inhibition of protein synthesis in the retinal pigment epithelium by chloroquine. Investigative Ophthalmology & Visual Science.

[19] Easterbrook M (1992). Long-term course of antimalarial maculopathy after cessation of treatment. Canadian Journal of Ophthalmology. Journal Canadien d'Ophtalmologie.

[20] Bergholz R, Schroeter J, Rüther K (2010). Evaluation of risk factors for retinal damage due to chloroquine and hydroxychloroquine. Brith J Ophthalmol.

[21] Rodriguez-Padilla JA, Hedges TR 3rd, Monson B, Srinivasan V, Wojtkowski M, Reichel E, Duker JS, Schuman JS, Fujimoto JG (2007). High-speed ultra-high-resolution optical coherence tomography findings in hydroxychloroquine retinopathy. Arch Ophthalmol.

[22] Yılmaz O, Saatcı AO (2009). Antimalaryal ilaç kullanan hastalarda retina sinir lifi kalınlığının değerlendirilmesi. J Retina-Vitreous.

[23] Kan E, Yakar K, Demirag MD, Gok M (2018). Macular ganglion cell-inner plexiform layer thickness for detection of early retinal toxicity of hydroxychloroquine. Int Ophthalmol.

[24] Lee MG, Kim SJ, Ham DI, Kang SW, Kee C, Lee J, Cha HS, Koh EM (2014). Macular retinal ganglion cell-inner plexiform layer thickness in patients on hydroxychloroquine therapy. Invest Ophthalmol Vis Sci.

[25] Bulut M, Erol MK, Toslak D, Akidan M, Kaya Başar E, Çay HF (2017). A New Objective Parameter in Hydroxychloroquine-Induced Retinal Toxicity Screening Test: Macular Retinal Ganglion Cell-Inner Plexiform Layer Thickness. Arch Rheumatol.

[26] Uslu H, Gurler B, Yildirim A, Tatar MG, Aylin Kantarcı F, Goker H, Pehlevan HS, Colak HN (2016). Effect of Hydroxychloroquine on the Retinal Layers: A Quantitative Evaluation with Spectral-Domain Optical Coherence Tomography. J Ophthalmol.

[27] Sallam MA, Beltagi AS, Abdellatif MA, Awadalla MA (2021). Visual Impact of Early Hydroxychloroquine-Related Retinal Structural Changes in Patients with Systemic Lupus Erythematosus. Ophthalmologica.

[28] Aydın Kurna S, Kanar HS, Garlı M, Çakır N (2022). Evaluation of the role of spectral-domain optical coherence tomography in the early detection of macular and ganglion cell complex thickness changes in patients with rheumatologic diseases taking hydroxychloroquine. Photodiagnosis Photodyn Ther.

